# Phase II Metabolism of Asarone Isomers In Vitro and in Humans Using HPLC-MS/MS and HPLC-qToF/MS

**DOI:** 10.3390/foods10092032

**Published:** 2021-08-29

**Authors:** Lena Hermes, Janis Römermann, Benedikt Cramer, Melanie Esselen

**Affiliations:** Institute of Food Chemistry, University of Muenster, Corrensstraße 45, 48149 Muenster, Germany; lena.hermes@uni-muenster.de (L.H.); ja.roemermann@gmail.com (J.R.); cramerb@uni-muenster.de (B.C.)

**Keywords:** asarone isomers, human study, metabolites, asarone glucuronides

## Abstract

(1) Background: Metabolism data of asarone isomers, in particular phase II, in vitro and in humans is limited so far. For the first time, phase II metabolites of asarone isomers were characterized and human kinetic as well as excretion data after oral intake of asarone-containing tea infusion was determined. (2) Methods: A high pressure liquid chromatography coupled with quadrupole time-of-flight mass spectrometry (HPLC-qTOF-MS) approach was used to identify phase II metabolites using liver microsomes of different species and in human urine samples. For quantitation of the respective glucuronides, a beta-glucuronidase treatment was performed prior to analysis via high pressure liquid chromatography coupled with tandem mass spectrometry (HPLC-MS/MS). (3) Results: Ingested beta-asarone and *erythro* and *threo*-asarone diols were excreted as diols and respective diol glucuronide conjugates within 24 h. An excretion rate about 42% was estimated. *O*-Demethylation of beta-asarone was also indicated as a human metabolic pathway because a corresponding glucuronic acid conjugate was suggested. (4) Conclusions: Already reported *O*-demethylation and epoxide-derived diols formation in phase I metabolism of beta-asarone in vitro was verified in humans and glucuronidation was characterized as main conjugation reaction. The excretion rate of 42% as *erythro* and *threo*-asarone diols and respective asarone diol glucuronides suggests that epoxide formation is a key step in beta-asarone metabolism, but further, as yet unknown metabolites should also be taken into consideration.

## 1. Introduction

Alpha-asarone (aA) and beta-asarone (bA) are phenylpropanoids, whose chemical structures only differ in the conformation of the double bound of the respective C3 side chain. These compounds mainly occur in essential oils of *Acorus* species, which are widely disseminated in Europe, North America and East Asia [1,2]. The predominant class is *Acorus (A.) calamus* L. Nevertheless, asarone contents strongly vary depending on several factors such as country of origin, polyploidy and plant materials e.g., roots or leaves [3]. In Indian tetraploid varieties, bA is present at high amounts of up to 95%, whereas aA amounts are on average about 15% [4].

Besides flavoring properties and bioactivity, essential oils as well as dried rhizomes or leaves of calamus are mainly used in herbal teas, frozen desserts, yoghurts, alcoholic and non-alcoholic beverages or food supplements [5]. Traditional phytomedicine and cosmetics also represent significant human exposure routes [2,4,5]. Moreover, asarone isomers and calamus-derived preparations are considered to show positive effects on human health such as antioxidant, anti-inflammatory, antidepressant, anti-microbial, neuro-, chemo- and radioprotective properties that facilitate their use as phytopharmaceutical summarized in Das et al. [6] and Chellian et al. [7]. Besides all positive effects, these substances are also of toxicological concern. Among acute and chronic toxicity, hepatotoxic properties in rodents as well as cytotoxic, genotoxic and mutagenic effects in vitro are reported for parent compounds and several oxidative phase I metabolites [7,8,9,10,11]. Furthermore, DNA repair mechanisms are also activated in response to asarone-mediated genotoxic effects in cells [10].

Phase I metabolism of aA and bA is elucidated in liver microsomes of different species. The main metabolite of aA is reported as 3′-oxoasarone, which arises out of (*E*)-3′-hydroxyasarone (3′OH) via further oxidation steps, whereas bA is mainly metabolized via an epoxide intermediate to *erythro*- and *threo*-1′,2′-dihydroxyasarone (*erythro*- and *threo*-asarone diols) and 2,4,5 trimethoxy-phenylacetone (asarone ketone) [12,13,14]. Even though in vitro phase I metabolism has been deeply characterised in the literature, little is known so far about potential phase II conjugation of these compounds. Nonetheless, the intake of *A. calamus* oil preparations in a human intervention trial proposes a renal hydroxylated bA metabolite after glucuronidase treatment, emphasizing glucuronic acid conjugation as possible phase II pathway [15]. This metabolite is also observed in urine of rats after an intraperitoneal application of aA [16].

Currently, there is no human data available regarding toxicokinetic parameters. In studies with rodents, for aA, bioavailability of 34% up to 78% is reported, which is further enhanced by inhalation [7]. Plasma half-life times of 29 min for aA and 13 min for bA are found after intravenous treatment of rats [17]. Depending on application form, longer half-life periods for oral and inhalative aA application, with values of 65 min up to 95 min, are reported [17,18]. In blood serum of rats, the half-life value of approximately 54 min indicate that bA is rapidly excreted [19]. Furthermore, marginal oral bioavailability has been considered, due the hydrophilic character of the asarone isomers, summarized in Chellian et al. [7]. Nevertheless, both isomers are shown to cross the blood brain barrier [18,20].

So far, in the European Union a maximum level of 1 mg/kg for bA is defined for alcoholic beverages, and for food a recommended maximum of 0.1 mg/kg is given [5,21]. For calamus preparations, it is recommended to use diploid varieties, which contain almost no or little bA amounts. For herbal medicine products, an intake of 2 µg/kg body weight/day is temporarily acceptable [5,22]. aA is not further regulated by law in the European Union, while bA is classified as a genotoxic carcinogen, thus the margin of exposure approach is used for risk assessment and a value below 10,000 is considered a high priority [23]. Toxicokinetic and toxicodynamic data of the genotoxin bA have to be urgently improved for adequate risk evaluation.

In consideration of the limited metabolism data of asarone isomers, in this study microsomal phase II metabolites were characterized using the respective phase I metabolites. Moreover, a human study was performed to identify main renal phase II metabolites. Ten participants consumed a commercially available calamus-containing infusion and gave urine samples over a period of 48 h. Furthermore, the study provided new insights into the excretion kinetic over the observation period and the overall rate of excretion, which was calculated considering the ingested asarone amounts. A high-pressure liquid chromatography coupled with quadrupole time-of-flight mass spectrometry (HPLC-qTOF-MS) approach was used to identify new metabolites in microsome samples and in human urine. Kinetic and excretion data were generated using validated high-pressure liquid chromatography with tandem mass spectrometric (HPLC-MS/MS) detection for quantitation.

## 2. Materials and Methods

### 2.1. Chemicals and Reagents

All solvents used for sample dilution or chromatography were of LC-MS-grade and purchased from Carl Roth (Karlsruhe, Germany), Fisher Scientific (Schwerte, Germany) or Sigma-Aldrich (Steinheim, Germany), if not stated otherwise. Water was purified using a Purelab Flex 2 system (Veolia Water Technologies, Celle, Germany). Formic acid (FA), and magnesium chloride (MgCl_2_) were ordered from Merck (Darmstadt, Germany), tris(hydroxymethyl)aminomethane (TRIS) from VWR (Darmstadt, Germany) and ammonium hydrogen carbonate from Carl Roth (Karlsruhe, Germany). Uridine-5′-diphosphate glucuronic acid (UDPGA), glucose-6-phosphate-dehydrogenase (G6P-DH) and NADP+ were purchased from Sigma-Aldrich and *E*. *coli* beta-glucuronidase from Romer Labs GmbH (Butzbach, Germany). G6P was from AppliChem (Darmstadt, Germany).

Microsomes from the species pig and horse were isolated according to the protocol of LAKE 1987 and stored at −80 °C in storage buffer [24]. Human microsomes (Corning^®^ UltraPool™, Corning, NY, USA) were purchased from Corning Inc. (Durham, NC, USA).

bA, 3′OH and (Z)-asarone-1′,2′-epoxide (bAE) were kindly provided by our project partner from the University of Kaiserslautern (Germany). *Threo*- and *erythro*-asarone diols were isolated from a decomposed epoxide solution. Further information can be found in the literature [10]. Additionally, 7′-hydroxycoumarin and 4-methylumbelliferyl-β-D-glucuronide were purchased from Sigma-Aldrich (Steinheim, Germany). All analyte solutions were prepared in acetonitrile and stored at −20 °C.

### 2.2. In Vitro Experiments

Phase II metabolites were generated by incubation of liver microsomes (rat, pig, human) with the respective phase I metabolites 3′OH and bAE. Subsequently characterization was carried out by a HPLC-qTOF-MS approach. Experimental conditions were set for glucuronic and sulfuric acid conjugation and experimental settings are described below. As sulfuric acid conjugation was not successful, further sample preparation focuses on glucuronidation. Details for sulfuric acid conjugation are described in Supporting Information Table S1.

#### Sample Preparation

The method to simulate glucuronic acid conjugation by microsomes using phase I metabolites was conducted in accordance with WU et al., 2007 [25]. In each reaction mixture protein concentration was normalized to 1 mg/mL, independent of the microsome species. The reaction mixture consisted of 0.3 mM UDPGA, 0.4 mM MgCl_2_ and 0.1 mM 3′OH or bAE, respectively. The volume of each reaction mixture was filled with 83.4 mM Tris buffer to 200 µL. Microsomes were directly added before the reaction was started. Each tube was gently vortexed and incubated at 37 °C for 4 h while gently shaking. To stop the reaction, 400 µL acetonitrile were added to each tube and the samples were centrifugated at 4 °C for 5 min and 14,000× *g*. 150 µL of supernatant were diluted with 850 µL of water achieving a final concentration of acetonitrile of 12%, noting the starting conditions of the following HPLC-qTOF-MS method. A blank sample without analyte was used to distinguish analyte signals from matrix signals. Moreover, a second blank sample without liver microsomes was implemented to distinguish enzymatic from chemical reactions. Efficiency of the used microsomal systems was verified with the control 7′-hydroxycoumarin (100 µM).

### 2.3. Human Study

#### 2.3.1. Study Conditions and Subjects

Ten healthy enrolled participants (five females and five males, age 25.8 ± 4.0, BMI 23.8 ± 1.9) were informed about the aim and scope of the study and gave their written consent to the study conditions prior to their commencement. Samples and food diaries were equipped with a six-digit number and assigned to each participant randomly. The study was approved by the research ethical committee of the University Hospital Münster, Germany (File reference: 2020-002-f-S).

#### 2.3.2. Study Design

**Figure 1:**
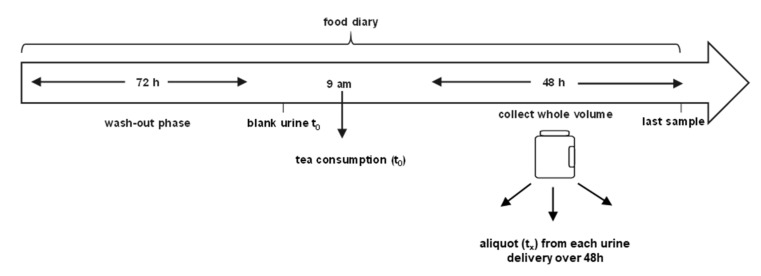
Study design including wash-out phase, diary keeping and urine collection after intake of 300 mL calamus tea infusion. Urine samples were collected for 48 h and total urine volume was determined. The participants collected a spot urine sample every time they urinated.

Study participants were not allowed to consume *A. calamus*-derived preparations or herbal products three days before intake of the tea infusion (wash-out phase), and wrote a food diary during the complete study progress (Figure 1). 

On day one, participants passed a morning urine sample as blank before calamus tea consumption. Tea preparation is described in (Section 2.3.3 “Calamus Tea selection”). All participants consumed at the same time 300 mL of the prepared tea infusion to ensure a consistent intake of asarone isomers. The tea infusion was consumed within half an hour and urine was collected for 48 h. The total urine volume over 48 h was determined by each participant by summarizing the volumes of each spot urine sample. Thereafter, an aliquot of 2 mL of each urine sample was collected for every time point (Figure 1). Urine samples were stored at 20 °C prior to sample preparation.

The total urine volume and the concentrations of *erythro*- and *threo*-asarone diols after beta-glucuronidase treatment was used to determine the excretion rate. Excretion values over 48 h were totalized and were compared with the amounts of *erythro*- and *threo*-asarone diols and bA in 300 mL of the consumed tea infusion. The average of all values for each participant was used as overall excretion rate (%).

The kinetic curve was determined using point in time of urination (aliquot of each urine delivery of each participant), given urine volume and concentrations of *erythro*- and *threo*-asarone diols in the urine samples over the period of 48 h. Time points were classified in two-hour blocks, except for the night hours (14–20 h) and the last 24 h, because in this period only a small number of samples was above the limit of quantitation (LOQ). The results were represented in a box-plot-whisker diagram. The average 50% of the data is located within the box, whose dimensions are defined by the lower and upper quartiles. The lowest and highest data points are shown by the whiskers if the values fall within the 1.5 interquartile range otherwise they are outliers and demonstrated as dots below and above the whiskers.

#### 2.3.3. Calamus Tea Selection

Calamus tea consisted of organically grown calamus roots, which have been dried and chopped prior to disposal. The used calamus infusion classified as food was analyzed within a previous product screening study [26]. Tea was prepared by weighing 48.5 g of dried calamus roots, infusing it with 3300 mL of boiling water and steeping for 15 min. For analysis of the asarone amount, an aliquot was filtrated using a 0.45 µM regenerated cellulose (RC) membrane (Phenomenex, Aschaffenburg, Germany), diluted with acetonitrile/0.1% formic acid in water (12/88, *v*/*v*) and analyzed by HPLC-MS/MS as reported previously [26]. 300 mL of the infusion contained 0.76 mg bA, 0.65 mg *erythro*-asarone diols and 1.38 mg *threo*-asarone diols. The amounts are in the mean of commercially available calamus infusions [26]. The fresh calamus tea was prepared by a separation of the rhizome from the *A. calamus* plant, which was bought in a local garden center (Vechta, Germany) The outer root layer was cleaned, and 3 g of calamus roots were crushed. Thereafter the roots were infused with 200 mL boiling water (100 °C). Steeping time, filtration, dilution and analysis were carried out as described above.

#### 2.3.4. Urine Sample Preparation

To 100 µL of each urine sample of each collection point, 100 µL of ammonium hydrogen carbonate (NH_4_HCO_3_) buffer (pH 6.6) were added which contained 6000 U/mL of beta-glucuronidase. The samples were incubated for 16 h at 37 °C by gently shaking. A volume of 200 µL acetonitrile were added to stop the enzyme reaction and after homogenization samples were centrifugated at 14,000× *g* for 5 min at 5 °C. Afterwards, the supernatant was diluted 1:10 with acetonitrile/0.1% formic acid in water (12/88, *v*/*v*) prior to HPLC-MS/MS or qTOF-MS analysis. Workability of beta-glucuronidase was verified by the 4-methylumbelliferyl-β-D-glucuronide converted to the highly fluorescent 4-methylum-belliferon and glucuronic acid. Fluorescence was measured at 365/440 nm.

### 2.4. Method Validation

The developed HPLC-MS/MS method for quantitation of *erythro*- and *threo*-asarone diols was evaluated with regard to the following parameters: linearity, limit of detection (LOD), LOQ, recovery as well as intraday and interday repeatability [27].

For quantitation of *erythro*- and *threo*-asarone diols in the urine samples, a matrix-matched calibration in blank urine of different volunteers was prepared. To that end, urine was processed as described in Section 2.3.4. and fortified with the analytes at concentrations of 0.25, 0.5, 1, 2.5, 5, 10, 25, 50 ng/mL, respectively. Linearity across the whole working range was verified by the Mandel’s fitting test and a coefficient of determination (R^2^) ≥ 0.995.

LOD and LOQ were determined using a matrix-matched approach in blank urine of different volunteers. Analytes were spiked in the following concentrations 0.05, 0.1, 0.25, 0.5, 1.0, 2.5, 5.0 and 10 ng/mL. Procedure was performed in triplicate. LOD and LOQ were determined using a Signal to Noise (S/N) approach receiving a S/N ratio of three for LOD and ten for LOQ.

For determination of recovery rates, blank urine was spiked with distinct analyte concentrations (0.25, 0.5, 1, 2.5, 5, 10, 25, 50 ng/mL) prior to sample preparation (Section 2.3.4). For calculation, the matrix-matched calibration was measured along with the matrix calibration and the slope of both calibration curves was compared. Each calibration point was prepared in triplicate.

The precision of the method is described by interday and intraday repeatability. Intraday repeatability was evaluated by preparing and analyzing one randomly chosen urine sample of one test person ten times. For interday repeatability one sample was prepared three times and repeatedly injected throughout the measurement of all urine samples of the study.

### 2.5. HPLC-MS/MS and HPLC-qTOF-MS Settings

Chromatographic separation for MS/MS analysis was achieved using a 1260 Infinity LC system (Agilent Technologies, Waldbronn, Germany). MS/MS analysis was performed using a QTrap^®^ 5500 mass spectrometer equipped with a Turbo V ion source and operated with Analyst software 1.6.2 (Sciex, Darmstadt, Germany). The obtained values for the MS parameters declustering potential (DP), collision energy (CE) and collision cell exit potential (CXP) were individually determined by infusing standard solutions into the MS system. MS parameters are as follows: Q_1_ (*m*/*z*), 225; Q_3_ (*m*/*z*) Q_N_ (quantifier transition)/Q_L_ (qualifier transition), 193/167; declustering potential (DP), 104; collision energy (CE) Q_N_/Q_L_, 18/23; CXP (V), 11. Retention time (RT): 5.39 min for *erythro*-asarone diols and 5.69 min for *threo*-asarone diols. Further HPLC-MS/MS setup details are presented in Supporting Information Table S2.

Chromatographic separation for qTOF-MS analysis was achieved using a Bruker Elute system (Bruker, Bremen, Germany). Mass spectrometric analysis was carried out on a Bruker impact II qTOF system equipped with an ESI Apollo II ion source operated in positive and negative ionization mode, depending on the analyte of interest (Bruker, Bremen, Germany). For identification of phase II metabolites, a full scan mode within a mass range of *m*/*z* 50 to 1300 as well as Auto MS/MS scan modes, were used. Further HPLC-qTOF-MS setup details for the analysis of liver microsome samples, as well as urine samples are given in the Supporting Information Tables S3 and S4.

## 3. Results

### 3.1. Microsome Experiments

Incubation of the selected phase I metabolites 3′OH and bAE with pig liver microsomes resulted in formation of different glucuronic acid conjugates. For bAE, it is re-ported that this compound is not stable and hydrolyzes in aqueous solution, with a half-life between 2.4 min and 4 min to *erythro*- and *threo*-asarone diols and asarone ketone [13,28]. 

**Figure 2:**
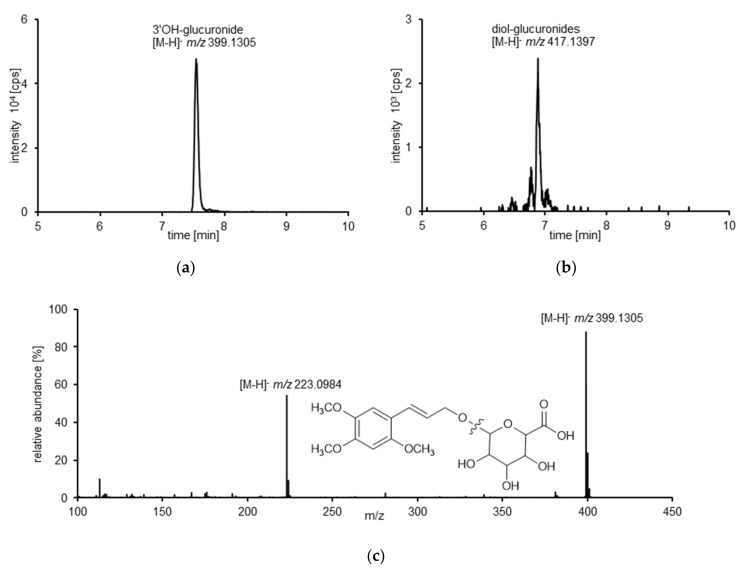
HPLC-qTOF-MS chromatograms after incubation of (**a**) 3′OH and (**b**) bAE in pig liver microsomes. Presented are the extracted-ion chromatogram (XICs) with the calculated mass of (**a**) *m*/*z* 399.1305 ± 0.01 for the 3′OH glucuronide and (**b**) *m*/*z* 417.1397 ± 0.01 for *erythro*- and *threo*-asarone diols-derived glucuronic acid conjugates. (**c**) HPLC-qTOF-MS spectrum of 3′OH glucuronide (*m*/*z* 399.1305 ± 0.01) with the respective structural formula and the suggested cleavage of the glucuronic acid majority to *m*/*z* 223.0984.

Consequently, incubation of bAE with microsomes resulted in diol-derived glucuronic acid conjugates. The extracted ion chromatograms (XICs) with *m*/*z* 399.1297 for 3′OH glucuronide (Figure 2a) and *m*/*z* 417.1402 for *erythro*- and threo-asarone diols-derived glucuronic acid conjugates (Figure 2b) allowed the detection of two peaks with mass differences (∆m) of 0.5 ppm and 0.8 ppm to the calculated masses of [M–H]^−^. Figure 2c shows the qTOF-MS spectrum of the 3′OH-glucuronide. The fragment with *m*/*z* 223.0984 can be assigned to the loss of the glucuronic acid moiety and corresponds to the [M–H]^−^ of 3′OH (Figure 2c). Due to its low concentration, the spectrum of the *erythro*- and threo-asarone diol-glucuronides did not provide significant fragmentation data. Liver microsomes of human and horse were also used to investigate the phase II metabolism of both phase I metabolites (3′OH, bAE). The respective glucuronic acid conjugates were formed by all species but with slightly different turnover rates (data not shown). Detailed information about species-specific phase II-Metabolism has to be considered in subsequent analyses and are not in the scope of the presented investigations. Sulfuric acid conjugation was not observed at all, indicating that glucuronidation can be considered as the main metabolic phase II pathway in microsomes from all species.

### 3.2. Method Validation

**Figure 3:**
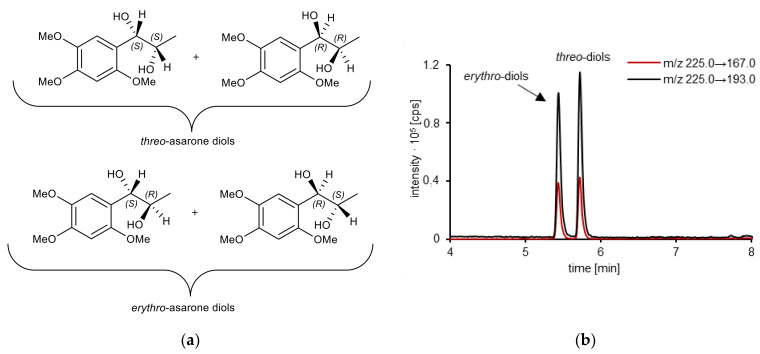
(**a**) Structural illustration of *erythro*- and *threo*-asarone diols and their stereochemistry. (**b**) HPLC-MS/MS chromatogram of a 1:10 diluted urine sample spiked with 5 ng/mL of erythro- and threo-asarone diols. Presented are the quantifier (*m*/*z* 225→193) and qualifier (*m*/*z* 225→167) SRM transition.

Method validation of the used HPLC-MS/MS method was performed prior to analysis of the urine samples from the human study. As *erythro*- and *threo*-asarone diols were found to be the dominant metabolites in urine after beta-glucuronidase treatment, quantitation of these compounds with a matrix-matched calibration in blank urine was performed. *Erythro*- and *threo*-asarone diols are diastereomers, which represent a pair of enantiomers, respectively (Figure 3a). 

Accordingly, with the used HPLC-MS/MS method, for the diastereomers *erythro*- and *threo*-asarone diols could be chromatographically separated, while the enantiomers coeluted. Figure 3b shows the analysis of one selected urine sample spiked with erythro and threo-asarone diols at a concentration of 5 ng/mL

From matrix-matched calibration, the validation parameters LOD and LOQ as well as linearity were determined. Linearity across the applied concentration range was confirmed by means of the Mandel’s fitting test as well as a R^2^ ≥ 0.995 for the analytes. The analytical precision via interday and intraday repeatability reached values of between 3% and 12% and recovery rates of 83% or 103% were determined. All values fall into an acceptable range considering the respective US Food and Drug Administration regulations [27]. The validation parameters are illustrated in Table 1.

### 3.3. Human Study

#### 3.3.1. Analysis of the Consumed Tea Infusion

The amounts of bA (0.76 mg) as well as *erythro*- (0.65 mg) and *threo*-diols (1.38 mg) in 300 mL of the consumed tea were used in total (2.79 mg) for calculation of the excretion rates.

#### 3.3.2. HPLC-MS/MS and qTOF-MS Analysis of Urine Samples

**Figure 4:**
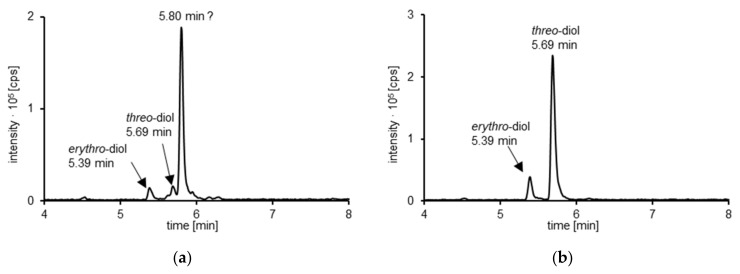
HPLC-MS/MS chromatogram of a randomly selected urine sample, which was given after consumption of a calamus tea infusion, (**a**) before; (**b**) after treatment with beta-glucuronidase.

Figure 4 shows HPLC-MS/MS chromatograms of an exemplary urine sample from one randomly selected participant before (a) and after beta-glucuronidase treatment (b), recorded in MRM-mode. The subsequently mentioned metabolism was observed in the urine of all participants with marginal differences in individual metabolite concentrations and excretion rates. The two peaks (5.39 and 5.69 min) represent the *erythro*- and *threo*-asarone diols, respectively, whereas the peak with a retention time of 5.80 min showing the same MRM transition could not be identified with the available standards (Figure 4a). No signal corresponding to 3′OH or asarone ketone was detected in all analyzed urine samples. Furthermore, no hints for a 3′OH glucuronide were found. However, after beta-glucuronidase treatment, the signal at 5.80 min disappeared, while the *erythro*-asarone diols peak (5.39 min) slightly and the *threo*-asarone diols peak (5.69 min) strongly increased (Figure 4b). These results suggest that the peak eluting at 5.80 min represents glucuronidated metabolites of the consumed asarone derivatives. 

**Figure 5:**
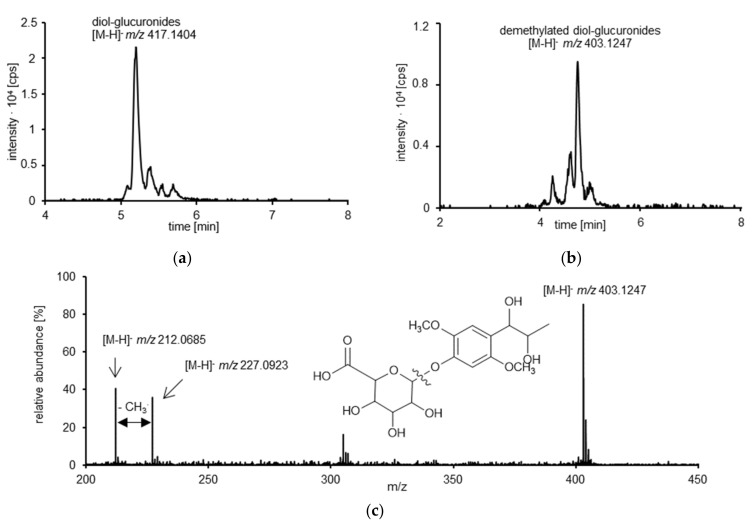
Exemplary HPLC-qTOF-MS chromatograms of a randomly selected urine sample before enzyme treatment. Presented are the extracted-ion chromatogram (XICs) with the calculated mass for (**a**) *erythro*- and *threo*-asarone diol glucuronides (diol-glucuronides, *m*/*z* 417.1404 ± 0.02) and (**b**) demethylated *erythro*- and *threo*-asarone diol glucuronides (demethylated diol-glucuronides, *m*/*z* 403.1247 ± 0.01). (**c**) HPLC-qTOF-MS spectrum of the *O*-demethylated *erythro*- and *threo*-asarone diol glucuronides (*m*/*z* 403.1247 ± 0.01) with the respective structural formula. The fragment *m*/*z* 227.0923 ± 0.02 corresponds to the *O*-demethylated metabolites after glucuronic acid cleavage. The loss of a further methyl group is shown by the exact mass of *m*/*z* 212.0685 ± 0.01.

To verify these findings and further to identify further new phase II metabolites, an untargeted HPLC-qTOF-MS approach was applied to human urine samples before beta-glucuronidase treatment. For the main peak, a mass of *m*/*z* 417.1404 ([C_18_H_26_O_11_–H]^−,^ ∆m: 0.2 ppm) supports the suggestion that *erythro*- and *threo*-asarone diol-glucuronides are potential phase II metabolites in humans (Figure 5a). Moreover, an unknown metabolite with an exact mass of *m*/*z* 403.1256 was detected in human urine. Based on a calculated *m*/*z* of 403.1256 for [C_17_H_24_O_11_–H]^−^, a mass difference of 1 ppm to the calculated mass suggested that also demethylated *erythro*- and *threo*-asarone diols-derived glucuronides were formed (Figure 5b). The recorded qTOF-MS spectrum supports our suggestions. The detected fragment ions of *m*/*z* 227.0923 are reported to arise due to the loss of the glucuronic acid moiety, and *m*/*z* 212.0685 with a further loss of a methyl group (Figure 5c).

As mentioned before, the calamus infusion used for the human study contains bA as well as *erythro*- and *threo*-asarone diols, thus the potential metabolization of bA to the identified phase II metabolites is not possible. Therefore, in a second proof of concept experiment, an infusion of fresh calamus roots was consumed by three participants and urine samples were collected as described above. It is reported that this tea infusion contains only bA (20 mg/kg) [26]. The analysis of these three urine samples showed that all characterized phase II metabolites are also excreted after single bA intake. Corresponding chromatograms are shown in Supporting Information Figure S1.

#### 3.3.3. Kinetic Studies and Excretion Rate Determination

**Figure 6:**
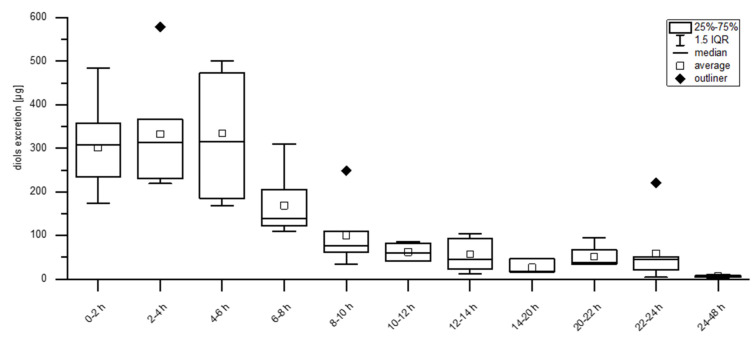
*Erythro*- and *threo*-asarone diols (diols) excretion kinetic [µg] of ten participants over a period of 48 h. Excretion is classified in two-hour blocks, except for the night hours (14–20 h) and the last 24 h, because the concentrations of the metabolites were mostly under the Limit of Quantification (LOQ).

Kinetic data and excretion rates were determined based on the analysis of the *erythro*- and *threo*-asarone diol peaks formed after beta-glucuronidase treatment, because the respective glucuronides were characterized as main human metabolites. The *O*-demethylated *erythro*- and *threo*-asarone diols-derived glucuronides were not included, because no corresponding reference compounds were available. Based on the above-mentioned finding, that an oral intake of bA also results in a renal excretion of *erythro*- and *threo*-asarone diols glucuronides, the overall amount of bA and *erythro*- and *threo*-asarone-diols was used to calculate total excretion rates. In sum, a total excretion of 42 ± 6% of all participants was determined. Kinetic data over a period of 48 h is shown in Figure 6. The respective phase II metabolites were rapidly excreted, with a maximum excretion between one and six hours. After 24 h only marginal amounts of *erythro*- and *threo*-asarone diols were further detected, thus the period between 24 h and 48 h was summarized in one bar.

## 4. Discussion

The scope of this work was the characterization of so far unknown asarone isomers-derived phase II metabolites in vitro and in humans, as well as a quantitative assessment of their excretion profile and kinetic in humans. In microsome experiments using the selected phase I metabolites bAE and 3′OH, new glucuronic acid conjugates, *erythro*- and *threo*-asarone diol glucuronides and 3′OH glucuronide, were identified based on their exact mass and mass spectrometric fragmentation pattern. After the intake of a bA- and *erythro*- and *threo*-asarone diols-containing tea infusion, signals for *erythro*- and *threo*-asarone diol glucuronides were detected, while no signals for further reported phase I metabolites e.g., 3′OH or asarone ketone were observed. However, in the presented human study, signals indicating *O*-demethylation reactions and respective glucuronic acid conjugation were found and suggest a further metabolic pathway.

In literature, a human renal glucuronic acid conjugate of hydroxylated aA is postulated after consumption of *A. calamus* oil without any further characterization [15]. The results of the conducted human study exclude the formation of a 3′OH-derived glucuronide. Side chain hydroxylation is hypothesized as main phase I metabolic pathway of aA [14]. Furthermore, the results suggest that side chain hydroxylation at position 3′ of bA does not occur in humans, even though it is a published pathway using liver microsomes of different species [13].

It is known that bAE rapidly decomposes to *erythro*- and *threo*-asarone diols and asarone ketone in aqueous solutions [13,28,29], hence glucuronic acid conjugation of *erythro*- and *threo*-asarone diols in liver microsomes after an incubation with bAE was detected. The consumed commercially available tea infusion contained besides bA also the *erythro*- and *threo*-asarone diols, thus raising the question as to whether the corresponding glucuronides are solely formed from the *erythro*- and *threo*-asarone diols present in the beverage. To answer this question, a second proof of the concept study with a calamus infusion of fresh non-dried roots, containing only the parent compound bA, was performed. However, the *erythro*- and *threo*-asarone diol-derived glucuronides were also found in a comparable pattern in human urine after consumption of this infusion. For the first time, these results explicitly emphasize that the epoxide-diol-pathway, which is identified as the main toxification pathway using liver microsomes [13], is also of special relevance in humans. In addition, these results are of high toxicological concern because the bAE is postulated as ultimate carcinogen [13]. Moreover, in mammalian cell systems bA-derived DNA adducts are identified and genotoxic effects of bAE are reported [9,28]. Considering the observed excretion rate of 42%, it is quite reasonable that the highly reactive epoxide intermediate is formed to a significant extent, which promotes its binding to macromolecules such as DNA or proteins. However, a fast repair of epoxide-derived genotoxic DNA-damage in liver tumor HepG2 cells and also a time-dependent decrease of DNA adducts in rat hepatocytes are reported [10,28]. Epoxide hydrolases catalyze the formation of less reactive dihydro-diol derivatives and they are suggested to play a major role in the detoxification of epoxides in vivo [30]. Nevertheless, missing data on the stability of bAE in vivo makes it difficult to assess potential further risks.

*O*-Demethylation was identified as a second metabolism pathway in humans because glucuronic acid conjugates of demethylated *erythro*- and *threo*-asarone diols were detected in human urine after intake of tea infusion of fresh or dried calamus roots, respectively. It is reported that O-demethylation, besides hydroxylation and epoxide formation plays a crucial role in microsomal metabolization of bA [13]. 

For the phenylpropenes elemicin, myristicin, and safrole several metabolites are characterized in human urine after nutmeg abuse or in urine samples of rats after drug administration. *O*-Demethylation and side chain hydroxylation are identified as main phase I reactions, whereas in phase II the functionalized metabolites are found to be partly conjugated to glucuronic acid or sulfuric acid [31]. Urinary recovery rates of the phenylpropenes estragole and eugenol of between 65–70% and 95% are reported and 50% of recovered eugenol is also excreted as glucuronic or sulfuric acid conjugates [32,33]. In contrast asarone derivatives were exclusively excreted as glucuronic acid conjugates, but with recovery rates below 50%. Regarding the excretion rate, it has to be mentioned that demethylated reference compounds are not available so far. Consequently, the detected demethylated asarone derivatives cannot be quantified after beta-glucuronidase treatment. Furthermore, no sulfonated metabolites were determined in human urine. These findings are in line with further in vitro investigations showing that the incubation of 3′OH and bAE with cytosolic fractions did not lead to sulfuric acid conjugates. It is also reported that sulfonation of 1′-hydroxy-estragole is less dominant in humans than in rodent species using respective liver S9-fractions [34]. Another work postulates 2,4,6-trimethoxycinnamic acid as a further metabolite of aA and bA in rat hepatocytes via LC-MS [35], which was not considered in this investigation.

The results show that, after calamus tea consumption by ten healthy participants, bA and *erythro*- and *threo*-asarone diols were quickly excreted within 24 h as respective glucuronides and, to a smaller extent, as non-conjugated *erythro*- and *threo*-asarone diols, reaching their maximum levels between 1 h and 6 h. A fast excretion and also a rapid glucuronidation is reported for the structure-related compound estragole after the intake of a fennel tea or the oral administration eugenol [34,36].

## 5. Conclusions

In sum, the results point out that uridine 5′-diphospho-glucuronosyltransferase-catalyzed conjugation reactions play a crucial role in phase II metabolism of asarone derivatives. This study confirms *O*-demethylation as an important metabolism pathway in humans for the first time. A fast renal excretion within 24 h is observed after calamus tea consumption. The recovery rate of only 42% emphasize that further work is still needed to characterize yet unknown human bA-related metabolites. A metabolization of bA via the epoxide-diol-pathway is suggested and should be considered for human risk assessment.

## Figures and Tables

**Table 1:** Method performance characteristics of the LC-MS/MS method used for quantitation of *erythro*- and *threo*-asarone diols in urine samples.

Substance	Linear Range [ng/mL]	LOQ [ng/mL]	LOQ [ng/mL]	Interday Repeatability [%]	Intraday Repeatability [%]	Recovery [%]
*erythro*-asarone diols	0.25–50	0.09	0.30	12.3	3.4	103
*threo*-asarone diols	0.25–50	0.06	0.25	8.5	8.3	83

## Data Availability

Data is contained within the article or Supplementary Materials.

## Supplementary Materials

The following are available online at https://www.mdpi.com/article/10.3390/foods10092032/s1, Figure S1: HPLC-qTOF-MS chromatogram of a selected urine sample after intake of fresh prepared bA-containing calamus tea, Table S1: Substances and concentrations used for phase II sulfonation experiments, Table S2: HPLC-MS/MS setup for the quantitation of *threo*- and *erythro*-asarone diols in urine samples, Table S3: HPLC-qTOF-MS setup for the screening of liver microsome samples for phase-II-metabolites originating from beta-asarone epoxide (bAE) and 3′hydroxyasarone (3′OH), Table S4: Characterization of unknown metabolites: Differences to HPLC-qTOF-MS setup presented in Table S2.
