# Supplementary material for: Phase II Metabolism of Asarone Isomers In Vitro and in Humans Using HPLC-MS/MS and HPLC-qToF/MS
Source: Foods. 2021 Aug 29;10(9):2032. doi: 10.3390/foods10092032 (PMC8467817; doi:10.3390/foods10092032)
Supplement: Supplementary file 1 [file foods-10-02032-s001.zip › foods-1319902-supplementary.pdf]

Phase II metabolism of asarone isomers *in vitro* and in humans using HPLC-MS/MS and HPLC-qToF/MS.

Supplementary Materials

Lena Hermes [1], Janis Römermann [1], Benedikt Cramer[1] and Melanie Esselen[1,*]

[1] University of Muenster, Institute of Food Chemistry, Corrensstraße 45, 48149 Muenster, Germany
* Correspondence: esselen@uni-muenster.de; Tel.: +49 251 8333874

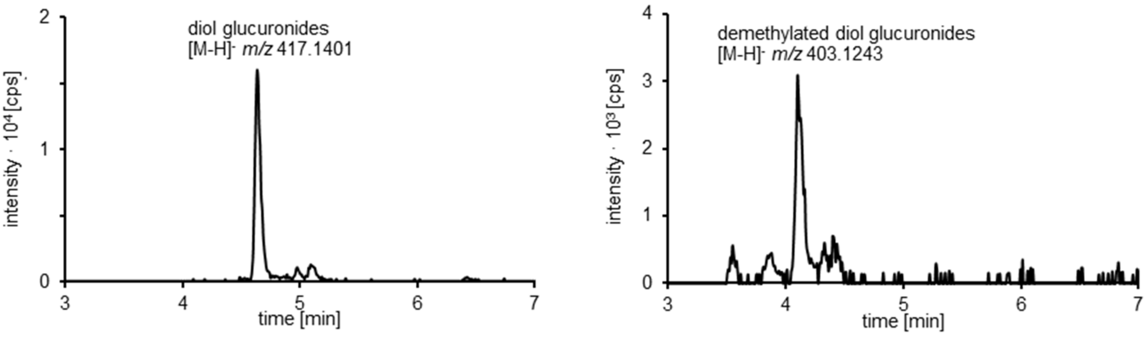

(**a**) (**b**)

Supporting information Figure S1. HPLC-qTOF-MS chromatogram of a selected urine sample after intake of fresh prepared bA-containing calamus tea. (**A**) *Erythro-* and *threo-*asarone diols-related glucuronides (diol gucuronides) with a calculated mass of m/z 417.1402 and (**B**) demethylated analogues with a calculated mass of m/z 403.1246.

Supporting Information Table S1. Substances and concentrations used for phase II sulfonation experiments. Sample preparation is according to 2.2.1. in the manuscript.

substance	concentration in reaction mixture
DTT (Merck, Darmstadt, Germany)a)	1.0 mM
PAPS (Sigma Aldrich, Steinheim, Germany)	0.1 mM
analyte	0.1 mM
liver cytosol	10 mg/mL
NaH_2PO_4 buffer (Merck, Darmstadt, Germany)	285 mM stock solution;<br>add to a final volume of 100 µL

[a)] DTT, 1,4-dithiotreitol; PAPS, adenosine-3′-phosphate-5′-phosphosulfate; NaH_2PO_4, sodium dihydrogen phosphate.

Supporting Information Table S2. HPLC-MS/MS setup for the quantitation of *threo*- and *erythro*-asarone diols in urine samples.

LC system	1260 Infinity LC system (Agilent, Waldbronn, Deutschland)		
column	ZORBAX Bonus RP (150 x 3.0 mm; 3.5 μm) (Agilent Technologies, Waldbronn, Deutschland); equipped with a C18 (4 x 2.0 mm) (Phenomenex, Aschaffenburg, Deutschland)		
flow rate	0.5 ml/min		
injection volume	10 μL		
column oven temperature	40 °C		
mobile phase	acetonitrile + 0.1% formic acid (A), H_2O + 0.1% formic acid (B)		
gradient	time [min]	A [%]	B [%]
	0	12	88
	1	12	88
	7	65	35
	7.01	100	0
	9	100	0
	9.01	12	88
	12	12	88
diverter valve	time [min]	position	
	0.00	Waste	
	3.00	MS	
	10.00	Waste	
Mass spectrometer	QTrap 5500 with Turbo V ion source Sciex, Darmstadt, Germany		
(polarity		positive	
spray voltage		4500 V	
source temperature		450 °C	
curtain gas (CUR)		1.03×10^5	
nebulizer gas (GS1)		2.41×10^5	
heater gas (GS2)		3.10×10^5	
scan event		MRM	

Supporting Information Table S3. HPLC-qTOF-MS setup for the screening of liver microsome samples for phase-II-metabolites originating from beta-asarone epoxide (bAE) and 3′hydroxyasarone (3′OH).

<table>
<tr><td>LC system</td><td colspan="3">Bruker Elute (Bruker, Bremen, Germany)</td></tr>
<tr><td>column</td><td colspan="3">ZORBAX Bonus RP (150 x 3.0 mm; 3.5 µm) (Agilent Technologies, Waldbronn, Deutschland); equipped with a C18 (4 x 2.0 mm) (Phenomenex, Aschaffenburg, Deutschland)</td></tr>
<tr><td>flow rate</td><td colspan="3">0.5 ml/min</td></tr>
<tr><td>injection volume</td><td colspan="3">10 µL</td></tr>
<tr><td>column oven temperature</td><td colspan="3">40 °C</td></tr>
<tr><td>mobile phase</td><td colspan="3">acetonitrile + 0.1% formic acid (A), H_2O + 0.1% formic acid (B)</td></tr>
<tr><td rowspan="7">gradient</td><td>time [min]</td><td>A [%]</td><td>B [%]</td></tr>
<tr><td>0</td><td>12</td><td>88</td></tr>
<tr><td>1</td><td>12</td><td>88</td></tr>
<tr><td>11</td><td>100</td><td>0</td></tr>
<tr><td>13</td><td>100</td><td>0</td></tr>
<tr><td>13.01</td><td>12</td><td>88</td></tr>
<tr><td>16</td><td>12</td><td>88</td></tr>
<tr><td rowspan="4">calibration</td><td>time [min]</td><td colspan="2">position</td></tr>
<tr><td>0.00</td><td colspan="2">Instrumental calibration</td></tr>
<tr><td>2.2</td><td colspan="2">MS</td></tr>
<tr><td>13.0</td><td colspan="2">Instrumental calibration</td></tr>
<tr><td>Mass spectrometer</td><td colspan="3">Bruker impact II qToF equipped with ESI Apollo II ion source</td></tr>
<tr><td>polarity</td><td colspan="3">positive/negative</td></tr>
<tr><td>capillary temperature</td><td colspan="3">250 °C</td></tr>
<tr><td>dry gas</td><td colspan="3">12 L/min</td></tr>
<tr><td>nebulizer gas</td><td colspan="3">4 bar</td></tr>
<tr><td>spray voltage</td><td colspan="3">4500 V/-3000 V</td></tr>
<tr><td>mass range</td><td colspan="3">m/z 50-1300</td></tr>
<tr><td>scan event 1</td><td colspan="3">full scan</td></tr>
<tr><td>spectra rate full scan</td><td colspan="3">1.9 Hz</td></tr>
<tr><td>scan event 2</td><td colspan="3">Auto MS/MS; No. of precursor: 1; active exclusion: exclude after 3 spectra; release after 0.3 min</td></tr>
<tr><td>spectra rate Auto MS/MS</td><td colspan="3">3.8 Hz</td></tr>
<tr><td>collision energy (Auto MS/MS)</td><td colspan="3">calculated from: m/z 100 = 15 eV, m/z 500 = 25 eV.m/z 1000 = 40 eV</td></tr>
<tr><td>XIC mass width</td><td colspan="3">± 0,01 Da</td></tr>
</table>

Supporting Information Table S4. Characterization of unknown metabolites: Differences to HPLC-qTOF-MS setup presented in Table 2.

gradient	time [min]	A [%]	B [%]
	0	12	88
	1	12	88
	7	65	35
	8	100	0
	11.50	100	88
	11.51	12	88
	14	12	88
calibration	time [min]	position	
	0.00	Instrumental calibration	
	2.2	MS	
	10	Instrumental calibration	
